# Perceived cognitive slowing and online health information seeking among older adults: motivational beliefs as pathways and community IT culture as a buffer

**DOI:** 10.3389/fpubh.2026.1875038

**Published:** 2026-06-26

**Authors:** Zian Fang, Zhenjun Zhao, Xianye Cao

**Affiliations:** 1 Xiangya School of Public Health, Central South University, Changsha, China; 2 School of Business, Central South University, Changsha, China; 3 School of Business, Hunan University of Science and Technology, Xiangtan, China; 4 School of Business Administration, Hunan University of Technology and Business, Changsha, China

**Keywords:** community IT culture, information processing speed, older adults, online health information seeking, outcome expectations, self-efficacy

## Abstract

**Background:**

Online health information seeking (OHIS) can support self-management and health decision-making among older adults, yet many still face barriers to digital health engagement. Perceived declines in information processing speed may constrain older adults’ ability and motivation to seek health information online, but the potential explanatory pathways and contextual conditions remain insufficiently understood. This study examined the associations between perceived information processing speed decline and older adults’ OHIS, with a focus on the roles of self-efficacy, outcome expectations, and community IT culture.

**Method:**

A cross-sectional survey was conducted among 295 adults aged 60 years and older. Data were collected using a structured questionnaire and analyzed with partial least squares structural equation modeling to test the proposed research model.

**Results:**

Perceived declines in information processing speed were negatively associated with self-efficacy but were not directly associated with outcome expectations or online health information seeking. Self-efficacy was positively associated with outcome expectations, and outcome expectations were positively associated with online health information seeking. Community IT culture moderated the association between perceived declines in information processing speed and self-efficacy, such that the negative association was weaker among older adults reporting stronger community IT culture. Bootstrapping further showed a significant sequential indirect association linking perceived declines in information processing speed to online health information seeking through self-efficacy and outcome expectations.

**Conclusion:**

Perceived declines in information processing speed were indirectly associated with online health information seeking through motivational beliefs rather than through a direct pathway. A supportive community IT culture may buffer the negative association between perceived processing speed decline and self-efficacy. These findings highlight the importance of strengthening self-efficacy and outcome expectations while fostering supportive community-level digital environments to promote digital health engagement among older adults.

## 1 Introduction

### 1.1 Background

In the context of rapid population aging and the increasing burden of chronic disease (1), online health information has become an important resource for supporting older adults’ health management and decision-making (1). Online health information seeking (OHIS) can reduce temporal and spatial barriers to accessing health resources and may support chronic disease self-management, preventive behaviors, and informed communication with health professionals (2). However, many older adults continue to face barriers to digital health engagement. Age-related functional limitations and cognitive changes may make it difficult for older adults to obtain, evaluate, and use online health information effectively (3). These barriers are not only individual challenges but also digital public health concerns, as they may limit older adults’ equitable access to health information and digital health resources.

Cognitive abilities are particularly relevant in online information environments, where users must search, compare, evaluate, and integrate information across multiple sources. Among these abilities, information processing speed has been identified as an important factor associated with older adults’ information-seeking behavior and task performance (4–7). The processing speed theory of cognitive aging further suggests that age-related slowing in cognitive operations may contribute to broader difficulties in complex information-processing tasks (8). Although prior studies have mainly treated information processing speed as an objective indicator, subjective cognitive aging research suggests that perceived cognitive changes may diverge from objective cognitive performance (9). Accordingly, this study focuses on perceived declines in information processing speed, defined as older adults’ subjective sense that processing, learning, understanding, or responding to information has become slower, more difficult, or more effortful (10). Because OHIS requires older adults to search for, evaluate, and integrate health information, such perceived declines may shape their judgments about whether they are capable of performing OHIS and whether the effort involved is worthwhile. However, existing research has mainly linked information processing speed to older adults’ digital technology use, information-seeking performance, or task outcomes (4–7), while paying limited attention to how perceived declines in information processing speed are associated with OHIS through motivational pathways. This gap limits our understanding of older adults’ real-world digital health engagement and makes it difficult to identify effective targets for digital public health interventions.

Social cognitive theory (SCT) offers a theoretical lens for addressing this question. SCT emphasizes that self-efficacy and outcome expectations are proximal determinants of behavior (11, 12). Within this framework, perceived declines in information processing speed may be understood as a form of negative cognitive and affective arousal that shapes older adults’ motivational beliefs (13), including their confidence in seeking online health information and their expectations about the benefits of doing so. Thus, self-efficacy and outcome expectations may constitute motivational pathways through which perceived declines in information processing speed are associated with OHIS.

SCT also emphasizes that environmental conditions can shape personal beliefs and behavior (11, 12). In China, communities are important settings in which older adults access, learn, and use digital technologies (14, 15). A supportive community IT culture may provide social encouragement, shared norms, peer learning opportunities, and a favorable environment for digital participation, making it a potentially modifiable environmental resource for digital public health interventions. However, although communities have been recognized as important contexts for older adults’ digital engagement, limited evidence explains how community IT culture may operate as a buffer against perceived cognitive challenges in OHIS.

To address these gaps, this study examines the associations between perceived declines in information processing speed and older adults’ online health information-seeking intention. Specifically, we investigate whether self-efficacy and outcome expectations serve as motivational pathways linking perceived declines in information processing speed to OHIS intention, and whether community IT culture buffers the negative association between perceived declines in information processing speed and these motivational beliefs. By integrating cognitive appraisal, motivational beliefs, and community-level digital context, this study aims to provide evidence for promoting older adults’ digital health engagement from a public health perspective.

### 1.2 Literature review and theoretical foundation

#### 1.2.1 Factors influencing older adults’ OHIS

Factors shaping older adults’ online health information seeking (OHIS) can be organized into three broad domains: technology-related, environment-related, and individual-related. Recent mixed-methods evidence further shows that older adults’ OHIS is not static, but develops into distinct behavioral patterns shaped by both technological, environmental and individual factors (16). At the technology level, one line of analysis focuses on attributes of digital technologies, such as perceived usefulness and perceived ease of use (17, 18); another emphasizes characteristics of the health information itself, including source credibility (19) and perceived reliability (20). Recent evidence further confirms the importance of online health information credibility, showing that older adults’ OHIS is associate with their trust in online health information and their perceived ability to evaluate the credibility of such information (21). These technology-related factors collectively reflect older adults’ expectations regarding the potential tangible benefits of OHIS. This is because such factors not only influence the cost and difficulty of the search process but also determine whether the information obtained can be understood, trusted, and transformed into actionable health advice.

Among environmental factors, social support positively influences older adults’ OHIS (22). Prior research further suggests that professional support and intergenerational support can also facilitate health information seeking in specific contexts (23, 24). Evidence suggests that the role of social support often does not directly promote information seeking behavior, but rather, through providing training and education, emotional encouragement, and experience demonstration, social support promotes such behavior through enhancing the self-efficacy of older adults (22, 25).

At the individual level, capacity-related factors constitute a salient set of determinants of older adults’ OHIS, as they reflect whether older adults are able, or believe they are able, to search for, process, and use online health information. A robust stream of research has examined capacity-related factors that capture older adults’ actual or perceived ability to engage in online health information seeking, including self-efficacy (18), health literacy (26), and eHealth literacy (27, 28). Work in this area has demonstrated that older adults with greater capabilities are more likely to seek online health information (3). These findings suggest that older adults’ OHIS is shaped not only by access to online information, but also by their judgments of whether they can complete the cognitive and digital tasks involved in OHIS.

Age-related characteristics may further shape these capacity-related experiences and represent a key dimension distinguishing older adults from other populations. Such characteristics, including physical functional limitations, vision impairment, and illness conditions, have been shown to impose constraints on older adults’ OHIS (3, 29). Moreover, subjective perceptions of aging-related cognitive changes have been shown to influence older adults’ digital engagement and motivational beliefs, such as technology anxiety, self-efficacy, and perceived usefulness (30, 31). In the context of OHIS, perceived declines in information processing speed may be particularly relevant because online health information seeking requires older adults to search, compare, evaluate, and integrate health information across digital sources. However, prior OHIS research has paid limited attention to this specific subjective age-related cognitive factor and to the motivational mechanisms through which it may be associated with older adults’ OHIS.

Taken together, prior research suggests that technological, environmental, and individual determinants of older adults’ OHIS are not merely isolated antecedents, but may operate through more proximal motivational beliefs. Technology-related factors may shape whether OHIS is perceived as useful and manageable; environmental factors may provide instruction, encouragement, and social support; and individual factors may influence whether older adults believe they are capable of searching for online health information. These diverse determinants can therefore be organized around two proximal psychological questions: whether older adults believe they can perform OHIS and whether they expect OHIS to produce beneficial outcomes. These two questions correspond closely to self-efficacy and outcome expectations, two central constructs in SCT. Thus, SCT provides an appropriate theoretical lens for explaining how aging-related characteristics and environmental conditions are translated into older adults’ OHIS through motivational mechanisms.

#### 1.2.2 Social cognitive theory

Social cognitive theory (SCT) offers a comprehensive framework for understanding how individual behavior emerges, changes, and is maintained within social contexts (11, 12). A core concept within SCT is the principle of triadic reciprocal determinism, which posits that behavior is not shaped unidirectionally by personal factors or environmental influences; rather, it arises from the dynamic interplay among personal factors, the environment, and behavior itself. Accordingly, SCT has been widely applied to examine individuals’ online health information behaviors, including seeking (24, 32, 33), sharing (34, 35), and fact-checking (36).

Several established frameworks, such as the Risk Information Seeking and Processing (RISP) model, the Planned Risk Information Seeking Model (PRISM), and the Theory of Motivated Information Management (TMIM), have been developed to explain health and risk information behaviors (37–39). For example, these frameworks have been widely applied to understand information seeking related to cancer risks, infectious disease outbreaks, and other health threats, where risk perception, information insufficiency, uncertainty, and affective responses serve as key motivational drivers (40–42). However, this study addresses a different theoretical question. Rather than focusing on a particular health risk or uncertainty episode, it examines how older adults’ perceived decline in information-processing is associated with OHIS through motivational beliefs, and how a supportive community IT culture may condition this process. SCT is therefore particularly suitable because it explicitly links personal factors, environmental conditions, cognitive-motivational beliefs, and behavior within a unified explanatory framework.

Social cognitive theory posits two key personal factors of behavior: self-efficacy and outcome expectations (11, 12). Its central proposition is that individuals are more likely to initiate and persist in an action when they believe they can perform it successfully and expect that doing so will yield beneficial outcomes. This relationship has been empirically supported in the context of technology adoption and information behavior (35, 43, 44). Recent studies on older adults’ digital health engagement provide further support for this proposition. Liu, Luo (45) showed that self-efficacy and outcome expectations facilitate older adults’ learning and use of digital technologies for health management, while Zhang, Xie (46) found that digital technology self-efficacy promotes older adults’ willingness to share health-tracking data. Bandura (11) identified four primary sources of self-efficacy and outcome expectancies: mastery experiences, vicarious experiences, verbal persuasion, and emotion arousal. Subsequent research further indicates that physiological arousal can shape beliefs about both self-efficacy and outcome expectations (47, 48). In the context of older adults’ engagement with digital technologies, the formation of self-efficacy and outcome expectations is similarly grounded in these pathways (13, 49). Importantly, age-related physiological changes, such as worsening memory and slowing reactions, can undermine older adults’ technology-related self-efficacy (13). This evidence provides a robust theoretical foundation for explaining how age-specific changes influence older adults’ technology-related behaviors.

Bandura (11, 12) also highlighted the importance of the social environment. Extant research has examined how social environments shape individual behavior from three perspectives. First, the social environment directly influences behavior. For example, in online health communities, users’ interactions with others and engagement with information can directly influence their health information exchange behaviors (34). Second, the social environment influences behavior indirectly by shaping individuals’ self-efficacy and outcome expectations. For instance, a supportive community climate can promote users’ knowledge-sharing intentions by enhancing self-efficacy and outcome expectations (50). Similarly, school IT culture positively influences students’ self-efficacy in using digital technologies (51). Further evidence from older adults’ digital health contexts suggests that community participation and family-provided digital feedback can both strengthen older adults’ self-efficacy in digital engagement (52, 53). Third, the social environment can condition the development of self-efficacy and outcome expectations, thereby playing a moderating role in the expectation beliefs formation process (54). In China, community settings constitute a primary context for older adults’ everyday lives. When learning digital technologies, older adults often receive practical guidance and vicarious experiences not only from younger family members but also from peers (55). Community-provided platforms and spaces further facilitate older adults’ acquisition of digital skills. Accordingly, this study conceptualizes community IT culture as a key social environmental factor and investigates how it shapes the development of older adults’ self-efficacy.

Taken together, SCT is particularly appropriate for this study because it captures the distinctive conditions under which older adults engage in OHIS. For older adults, OHIS is not only an information-seeking behavior, but also a cognitively and digitally demanding activity shaped by aging-related changes, limited digital experience, and concerns about making mistakes (3). Because behavioral intention is widely recognized as an important antecedent of actual behavior, this study focuses on older adults’ intention to engage in OHIS as a key indicator of their readiness to perform such behavior. In this context, perceived declines in information processing speed represent an age-related personal cognitive cue that may influence older adults’ self-efficacy and outcome expectations. Moreover, Compared with younger adults, older adults often face greater barriers to digital engagement and therefore rely more heavily on family, peer, and community-based support, making community IT culture a salient environmental condition (56). Prior studies have also applied SCT to explain older adults’ technology use and digital health-related behaviors (43, 49). Accordingly, this study adopts SCT to examine how perceived declines in information processing speed are associated with older adults’ OHIS intention through self-efficacy and outcome expectations, and how community IT culture conditions this process.

### 1.3 Hypothesis development

**Figure 1:**
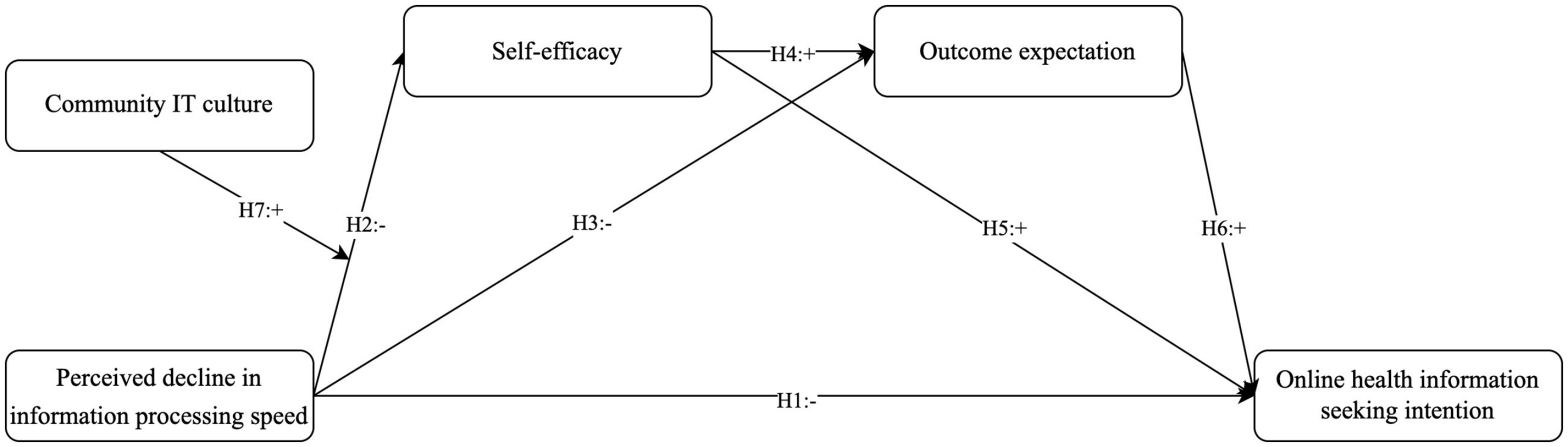
Research model.

This study develops a research model based on social cognitive theory and literature on online health information seeking among older adults, as illustrated in Figure 1.

#### 1.3.1 The impact of perceived decline in information processing speed

When older adults perceive declines in their information processing speed, online health information seeking may become a more cognitively demanding activity. OHIS requires older adults to scan search results, compare information across sources, evaluate credibility, and integrate health information into personally meaningful judgments (7). Perceived declines in information processing speed may therefore be associated with lower perceived efficiency in completing these tasks and greater concern about cognitive overload (6, 57). Older adults who perceive such declines may also feel less able to keep pace with rapidly updated online information, which may increase uncertainty and perceived risk, such as missing important information or relying on inaccurate content (58). This burden can prompt avoidance of OHIS (59). Therefore, we hypothesize:

H1: Perceived declines in information processing speed are negatively associated with older adults’ online health information seeking intention.

Perceived declines in information processing speed may also be associated with older adults’ motivational beliefs during online health information seeking. According to SCT, self-efficacy and outcome expectations are proximal cognitive antecedents of behavior. When older adults perceive declines in their information processing speed, they may regard multi-step search activities, such as generating search terms, evaluating search results, and navigating across webpages, as more difficult to complete efficiently. Such perceptions may be associated with less successful search experiences, including difficulty locating desired information or evaluating information credibility, which may in turn be linked to lower self-efficacy and less favorable outcome expectations (49). In addition, perceived declines in information processing speed may constitute a form of negative cognitive and affective arousal. Older adults who perceive such declines may experience greater anxiety or frustration during OHIS, particularly when they encounter complex health information, misinformation risks, privacy concerns, or search failures (3). Prior research suggests that negative cognitive and affective states can be associated with lower self-efficacy and less favorable outcome expectations (47, 48). Therefore, we hypothesize:

H2: Perceived declines in information processing speed are negatively associated with older adults’ self-efficacy.

H3: Perceived declines in information processing speed are negatively associated with older adults’ outcome expectations.

#### 1.3.2 The impact of self-efficacy and outcome expectation

Self-efficacy refers to an individual’s judgment of their capability to successfully perform a task (12). According to SCT, when individuals believe they can accomplish a task, they are more likely to anticipate that engaging in the behavior will generate benefits; in contrast, when they perceive the task as difficult or beyond their abilities, they are less likely to expect favorable outcomes, even if the behavior is objectively beneficial (11). In the context of OHIS, older adults with high self-efficacy may have higher expectations regarding the benefits of online health information (24). Beyond shaping outcome expectations, self-efficacy exerts a direct influence on behavior. Individuals with higher self-efficacy are more inclined to initiate the behavior, invest greater time and cognitive resources, and persist in the face of difficulties (11, 60). In contrast, individuals with lower self-efficacy will avoid target behaviors. For example, older adults with lower self-efficacy tend to avoid or resist digital health services (61), which constrains their engagement in OHIS (3). Accordingly, we hypothesize:

H4: Self-efficacy is positively associated with older adults’ outcome expectations.

H5: Self-efficacy is positively associated with older adults’ online health information seeking intention.

Outcome expectations refer to an individual’s belief about the potential benefits of engaging in a given behavior (12). In the context of OHIS, outcome expectations function as a central motivational mechanism for older adults because these expectations shape their cost–benefit evaluation of whether searching is worth the time and effort (24). When older adults expect that online health information will be useful, such as in improving their understanding of health conditions, supporting treatment decisions, or enhancing self-management, they are more inclined to initiate and sustain information seeking (17). This tendency is particularly pronounced among those in poorer health, who typically face more health-related challenges and have stronger health needs (49). In contrast, if prior experiences are unsatisfactory and fail to deliver the anticipated benefits, older adults are less likely to repeat the behavior (3). Therefore, we hypothesize:

H6: Outcome expectations are positively associated with older adults’ online health information seeking intention.

#### 1.3.3 The moderating effect of community IT culture

Community IT culture reflects shared norms and value orientations regarding the use of digital technologies within a community, such as smartphones and other digital tools (51). It may be reflected in the extent to which community members recognize the importance of digital technologies in daily life and encourage their adoption and diffusion. In this sense, community IT culture represents a salient social environment that may shape older adults’ cognitive -motivational responses, as well as their affective responses, during online health information seeking. This argument is consistent with risk information behavior theories, such as RISP and PRISM, which conceptualize health-related information seeking as a process shaped by both cognitive evaluations, such as perceived information-gathering capacity and perceived control, and affective responses, such as worry, anxiety, and uncertainty (37, 38). A supportive community IT culture may provide older adults with normative and motivational cues that digital technology use is common, manageable, and learnable (56). Empirical evidence further suggests that supportive digital and social environments can alleviate older adults’ health information anxiety and technophobia (52, 62). For older adults who perceive declines in information processing speed during OHIS, exposure to peers who use digital technologies and encouragement from community members may help them interpret such difficulties as manageable challenges rather than as signs of irreversible decline. By reducing uncertainty or anxiety associated with complex online health information, this supportive context may therefore be associated with greater confidence in their ability to acquire digital skills, search for online health information, and remain engaged in digital society (43). In contrast, in communities with weaker IT culture, older adults may have fewer opportunities to observe successful technology use, receive encouragement, or participate in peer learning. Under these conditions, perceived declines in information processing speed may be more strongly associated with lower self-efficacy, because older adults may interpret search difficulties, unfamiliar interfaces, or information evaluation problems as evidence that they are unable to use digital technologies effectively. Therefore, we hypothesize:

H7: Community IT culture moderates the association between perceived declines in information processing speed and self-efficacy, such that the negative association is weaker when community IT culture is stronger.

## 2 Materials and methods

### 2.1 Research design, data collection, and sample

This study adopted a cross-sectional survey design. Data were collected in February 2024 through Credamo, an online survey platform in China. Credamo maintains a user panel of more than 3 million individuals. According to information provided by the platform, the distribution of its respondent panel is broadly aligned with the overall structure of Chinese internet users, which provides a basis for recruiting a diverse online sample of older adults.

This study has been performed in accordance with the Declaration of Helsinki. Approval was granted by the Academic Committee of Business School at Central South University, and the study complied with ethical standards (ID: CSUBS20240110). All the participants have signed an informed consent.

A total of 509 completed questionnaires were initially collected. Because this study focused on older Chinese adults’ intention to seek health information online, 99 questionnaires from respondents younger than 60 years were excluded. In addition, an attention-check item was embedded in the questionnaire to identify inattentive responses. The item instructed respondents to select “strongly disagree.” Questionnaires from respondents who failed this attention check were excluded, resulting in the removal of 115 questionnaires.

After these exclusions, 295 valid questionnaires were retained for the final analysis. The proposed model included five latent variables and 19 observed indicators. Following Westland’s (63) sample size recommendation for structural equation modeling, the minimum required sample size was estimated as 112. To assess whether the sample size was adequate for the PLS-SEM analysis, an *a priori* power analysis was conducted using G*Power based on the maximum number of predictors pointing to an endogenous construct. With five predictors, a medium effect size of *f*^2^ = 0.15, *α* = 0.05, and statistical power of 0.80, the minimum required sample size was 92. The final sample size of 295 therefore exceeded this requirement, indicating adequate statistical power for estimating the proposed PLS-SEM model. Therefore, the final sample size exceeded the minimum requirement and was considered adequate for the planned partial least squares structural equation modeling analysis. Among the valid respondents, age ranged from 60 to 86 years, with a mean age of 64.705 years (SD = 4.591). Detailed demographic characteristics are presented in Table 1.

**Table 1:** Demographic characteristics.

Variable	Item	Frequency	Percentage
Gender	Male	155	52.5%
	Female	140	47.5%
Education	Elementary school or lower	29	9.8%
	Junior high school	48	16.3%
	Senior high school	90	30.5%
	College degree	40	13.6%
	Bachelor’s degree	80	27.1%
	Master’s degree or higher	8	2.7%
Experience (year)	Under 2 years	26	8.8%
	2–6 years	109	36.9%
	More than 6 years	160	54.2%

### 2.2 Measurement instrument

All constructs in the study model were measured using items adapted from prior research. To fit the context of online health information seeking among older adults in China, we modified the wording of selected items while preserving their original conceptual meanings. Perceived declines in information processing speed were measured using items adapted from the perceived processing speed difficulty scale developed by Fasbender, Gerpott (10). Consistent with the subjective nature of this construct, the items captured respondents’ self-reported experiences of increased difficulty, slowness, and effortfulness when processing information. Self-efficacy was measured using items adapted from Niehaves and Plattfaut (64). Outcome expectations were assessed using items adapted from Mou, Shin (65). Community IT culture was measured based on the approach reported by Wei, Teo (51). Online health information seeking intention was measured using items adapted from Li and Wang (66). All items were rated on a five-point Likert scale ranging from 1 = strongly disagree to 5 = strongly agree.

Because the survey was administered in Chinese, we used a translation and back-translation procedure to ensure semantic equivalence. The translated items were further reviewed and discussed by the research team to ensure conceptual consistency and contextual appropriateness for Chinese older adults’ online health information seeking. Before formal data collection, a pilot survey was conducted to assess whether older adults could understand and complete the questionnaire accurately. Fifteen older adults with prior experience using smartphones were invited to pretest the instrument. Participants provided feedback on item clarity, wording difficulty, and completion problems. Based on their feedback, ambiguous expressions were revised, and uncommon or difficult terms were replaced with wording more consistent with older adults’ comprehension habits. The full questionnaire is provided in Appendix A.

## 3 Results

### 3.1 Measurement model

We employed partial least squares-based SEM (PLS-SEM) with SmartPLS (version 4.0) to analyze the data. Although SCT is a well-established theoretical framework, the present study provides a context-specific extension of SCT by examining how perceived declines in information processing speed associate are associated with older adults’ online health information seeking through self-efficacy and outcome expectations, and by testing community IT culture as a contextual boundary condition. This extension results in a complex latent-variable model involving multiple constructs, two mediating mechanisms, and one moderating effect. Given the complexity of the proposed model and the non-normal distribution of the data, PLS-SEM with bootstrapping procedures was considered appropriate for testing the hypothesized direct, indirect, and moderating effects (67–70).

We assessed the measurement model for reliability, convergent validity, and discriminant validity.

As shown in Table 2, the Cronbach’s *α* and composite reliability (CR) value were all above the 0.7 benchmark, factor loading for all items exceeded 0.70, and each construct’s average variance extracted (AVE) was greater than 0.50 (71). This establishes the reliability and convergent validity of the constructs. The results for discriminant validity are presented in Table 3. The square root of each construct’s AVE exceeded its correlations with other constructs (71), and all HTMT values were below 0.9 (72). In addition, item cross-loadings were examined as an additional assessment of discriminant validity. As reported in Appendix B, all indicators loaded more strongly on their intended constructs than on any other constructs (73). Taken together, these results provide convergent evidence that the constructs in our measurement model possess adequate discriminant validity.

**Table 2:** Results of reliability and convergent validity.

Construct	Item	Factor loading	Cronbach’s *α*	CR	AVE
Perceived decline in information processing speed	PD1	0.759	0.927	0.955	0.773
	PD2	0.894			
	PD3	0.892			
	PD4	0.922			
	PD5	0.918			
Self-efficacy	SE1	0.790	0.819	0.846	0.732
	SE2	0.898			
	SE3	0.875			
Outcome expectation	OE1	0.876	0.897	0.897	0.764
	OE2	0.886			
	OE3	0.868			
	OE4	0.866			
Community IT culture	CC1	0.912	0.801	0.882	0.712
	CC2	0.727			
	CC3	0.882			
OHIS intention	OHISI1	0.869	0.854	0.857	0.696
	OHISI2	0.823			
	OHISI3	0.821			
	OHISI4	0.821			

**Table 3:** Results of discriminant validity.

Construct	PD	SE	OE	CC	OHISI
PD	0.879	0.444	0.204	0.256	0.104
SE	−0.393	0.856	0.585	0.309	0.392
OE	−0.201	0.517	0.874	0.502	0.734
CC	−0.258	0.269	0.425	0.844	0.581
OHISI	−0.079	0.336	0.644	0.465	0.834

Below the diagonal: standardized correlations among the constructs; On the diagonal (in bold): Square roots of the AVE; above the diagonal (in italics): Heterotrait–Monotrait ratio (HTMT) values.

### 3.2 Common method bias

To assess common method bias, we conducted Harman’s one-factor test, which identified four latent factors, with the largest accounting for 35.59% of the variance, below the threshold of 40% (74). Following Liang, Saraf (75), we calculated each indicator’s variance substantively explained by the principal construct and by the common method factor. As shown in Table 4, the ratio of substantive to method variance is about 86:1, and most factor loadings are not significant. These results indicate that common method bias was not a significant concern for this study.

**Table 4:** Common method bias analysis.

Construct	Indicator	Substantive factor loading (R1)	R1^2^	Method factor loading (R2)	R2^2^
PD	PD1	0.972***	0.945	0.261***	0.068
	PD2	0.902***	0.814	0.013	0.000
	PD3	0.839***	0.704	−0.071	0.005
	PD4	0.817***	0.667	−0.135***	0.018
	PD5	0.890***	0.792	−0.038	0.001
SE	SE1	0.889***	0.790	−0.084	0.007
	SE2	0.877***	0.769	0.028	0.001
	SE3	0.806***	0.650	0.053	0.003
OE	OE1	0.861***	0.741	0.024	0.001
	OE2	0.901***	0.812	−0.018	0.000
	OE3	0.853***	0.728	0.017	0.000
	OE4	0.881***	0.776	−0.022	0.000
CC	CC1	0.781***	0.610	0.129**	0.017
	CC2	0.860***	0.740	−0.123	0.015
	CC3	0.904***	0.817	−0.019	0.000
OHISI	OHISI1	0.826***	0.682	0.062	0.004
	OHISI2	0.768***	0.590	0.098*	0.010
	OHISI3	0.875***	0.766	−0.087*	0.008
	OHISI4	0.868***	0.753	−0.076	0.006
AVG		0.862	0.745	0.001	0.009

***p < 0.001, **p < 0.01, *p < 0.05.

### 3.3 Structural model

**Figure 2:**
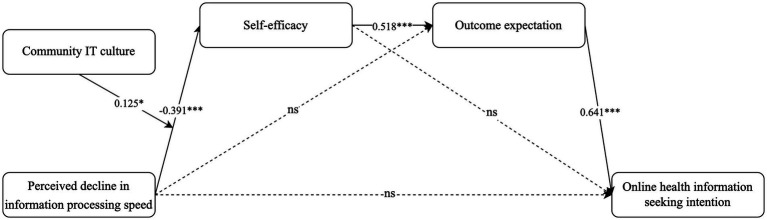
Results of structural model.

We assessed the structural model using a bootstrapping procedure with 5,000 resamples. The results are presented in Figure 2 and Table 5. The model explained 42.43% of the variance in online health information seeking. As supplementary evidence of approximate model fit, the standardized root mean square residual (SRMR) was examined. The SRMR value was 0.057, below the threshold of 0.08, indicating acceptable approximate fit (76). Age, gender, education level, and duration of smartphone use were included as control variables; none of these variables was significantly associated with online health information seeking intention.

**Table 5:** Hypothesis testing.

Hypothesis	Path coefficient	*p*-value	Results
H1: PD→OHIS	0.054^ns^	0.200	Not supported
H2: PD→SE	−0.391^***^	<0.001	Supported
H3: PD→OE	0.002^ns^	0.962	Not supported
H4: SE→OE	0.518^***^	<0.001	Supported
H5: SE→OHISI	0.032^ns^	0.616	Not supported
H6: OE→OHISI	0.646^***^	<0.001	Supported
H7: CC→(PD→SE)	0.125^*^	0.039	Supported

***p-value < 0.001, **p-value < 0.01, *p-value < 0.05, ns = not significant.

Perceived declines in information processing speed were not significantly associated with older adults’ outcome expectations or online health information seeking intention. However, perceived declines in information processing speed were negatively associated with self-efficacy. Thus, H2 was supported, whereas H1 and H3 were not supported. Self-efficacy was positively associated with outcome expectations but was not significantly associated with online health information seeking intention. Therefore, H4 was supported, whereas H5 was not supported. Outcome expectations were positively associated with online health information seeking intention, supporting H6.

Community IT culture significantly moderated the association between perceived declines in information processing speed and self-efficacy. Specifically, the negative association between perceived declines in information processing speed and self-efficacy was weaker among older adults reporting higher levels of community IT culture. Therefore, H7 was supported.

To examine the potential indirect association linking perceived declines in information processing speed to online health information seeking intention, we conducted a bootstrapped statistical mediation analysis with 5,000 resamples (see Table 6). The results showed a significant sequential indirect association between perceived declines in information processing speed and online health information seeking intention through self-efficacy and outcome expectations. Specifically, higher perceived declines in information processing speed were associated with lower self-efficacy; lower self-efficacy was associated with less favorable outcome expectations; and less favorable outcome expectations were associated with lower intention to seek health information online.

**Table 6:** Results of the mediating effect analysis.

Relationships	*β*	Boot SE	*p*-value	Bootstrap 95% CIs	
				LLCI	ULCI
Total effect of PD→OHIS	−0.088^ns^	0.053	0.098	−0.197	0.013
Total effect of PD→OE	−0.201^***^	0.046	<0.001	−0.292	−0.115
Total effect of SE→OHISI	0.366^***^	0.066	<0.001	0.233	0.495
Direct effect of PD→OHISI	0.054^ns^	0.042	0.200	−0.030	0.134
Total indirect effect of PD→OHISI	−0.142^***^	0.035	<0.001	−0.214	−0.076
PD→SE→OE	−0.203^***^	0.028	<0.001	−0.260	−0.153
PD→SE→OHISI	−0.012^ns^	0.025	0.620	−0.061	0.037
PD→OE→OHISI	0.001^ns^	0.028	0.962	−0.054	0.056
PD→SE→OE→OHISI	−0.131^***^	0.021	<0.001	−0.176	−0.092
SE→OE→OHISI	0.335^***^	0.048	<0.001	0.245	0.432

***p-value < 0.001, **p-value < 0.01, *p-value < 0.05, ns = not significant.

## 4 Discussion

### 4.1 Key findings

This study examined the associations between perceived declines in information processing speed and older adults’ OHIS intention, with a focus on potential motivational pathways and the moderating role of community IT culture. The findings showed that perceived declines in information processing speed were not directly associated with OHIS intention. Instead, they were indirectly associated with lower OHIS intention through lower self-efficacy and less favorable outcome expectations. In addition, stronger community IT culture attenuated the negative association between perceived declines in information processing speed and self-efficacy. These findings suggest that older adults’ OHIS intention may be associated not only with individual cognitive perceptions and motivational beliefs but also by the digital environment of the communities in which they live.

Compared with barriers emphasized in prior research, such as technology anxiety, low self-efficacy, and limited literacy (3), perceived declines in information processing speed may capture a more age-related cognitive concern in older adults’ digital health engagement. Although prior studies have examined functional limitations such as declining vision and deteriorating physical condition as barriers faced by older adults (77), this study focused on perceived age-related cognitive changes in the context of online health information seeking. We conceptualized perceived declines in information processing speed as a subjective cognitive self-perception that reflects older adults’ perceived difficulty in processing complex or novel online health information. The results suggest that such perceived declines in information processing speed are associated with OHIS intention through motivational beliefs rather than through a direct pathway. Specifically, older adults who reported greater perceived declines in information processing speed tended to report lower self-efficacy, which was associated with less favorable outcome expectations, and less favorable outcome expectations were associated with lower OHIS intention. For older adults, OHIS may be driven by multiple practical needs, such as managing chronic conditions, preparing for medical consultations, verifying health advice, or supporting everyday health decisions. Therefore, even when older adults perceive that processing online information has become slower or more effortful, they may still intend to seek online health information if they consider it relevant or necessary. In this sense, perceived declines in information-processing speed may not operate as a direct behavioral barrier; rather, their relevance for OHIS intention appears to depend on whether they are accompanied by self-efficacy and outcome expectations. This pattern is consistent with prior evidence suggesting that self-efficacy and outcome expectations are proximal motivational correlates of older adults’ OHIS (49).

Beyond the nonsignificant direct association between perceived declines in information-processing speed and OHIS intention discussed above, two other nonsignificant direct associations also warrant attention. First, perceived declines in information-processing speed were not directly associated with outcome expectations. This may be because perceived processing difficulty and outcome expectations reflect different evaluative judgments. Older adults may feel that processing online health information has become slower or more effortful, but this does not necessarily mean that they view OHIS as less useful for understanding health issues, managing illness, or supporting medical decision-making. Instead, perceived processing difficulty may be related to outcome expectations only when it is accompanied by lower self-efficacy: older adults who feel less capable of performing OHIS may also be less likely to expect favorable outcomes from it. Second, self-efficacy was not directly associated with OHIS intention after outcome expectations were considered. One possible explanation is that confidence in performing OHIS reflects perceived capability, whereas intention depends more closely on whether older adults regard OHIS as necessary and valuable (49). Thus, self-efficacy may correspond to OHIS intention primarily when it is linked to favorable outcome expectations.

The study further found that community IT culture moderated the association between perceived declines in information processing speed and self-efficacy. One possible interpretation is that a supportive community IT culture provides a positive digital climate, social encouragement, and favorable learning models. In such environments, older adults may be more likely to view digital technology use as common, manageable, and learnable, and may draw confidence from observing or interacting with people around them. This supportive context may be associated with a weaker negative association between perceived declines in information processing speed and self-efficacy. Unlike strategies that focus primarily on technical training or digital literacy improvement (25, 78, 79), our findings highlight the importance of building inclusive and supportive digital environments for addressing digital engagement challenges in an aging population. From a digital public health perspective, promoting older adults’ access to online health resources may benefit from attention not only to individual capabilities but also community-level environments associated with digital participation. This community-level perspective is particularly important because supportive digital environments are potentially modifiable through public health programs, community education, peer support, and age-friendly digital inclusion initiatives.

### 4.2 Theoretical contributions

This study contributes to the literature on older adults’ OHIS intention by introducing an aging-related cognitive perception, examining its potential motivational pathways, and identifying a community-level contextual boundary condition.

First, this study contributes to the literature on older adults’ OHIS by shifting attention from chronological age as a broad demographic variable to a more specific aging-related perception: perceived declines in information processing speed. Prior research has shown that older adults are less likely to seek online health information than younger adults (80, 81). However, age itself provides limited explanatory insight into which aging-related changes are associated with lower OHIS intention. By focusing on perceived declines in information processing speed, this study offers a more specific account of how older adults’ subjective perceptions of cognitive change are linked to digital health engagement. The findings indicate that perceived declines in information processing speed were not directly associated with OHIS intention but were indirectly associated with lower OHIS intention through lower self-efficacy and less favorable outcome expectations. This extends prior research that has primarily emphasized general predictors such as perceived usefulness, eHealth literacy, and social support (3), and highlights the importance of incorporating subjective aging-related cognitive perceptions into models of older adults’ OHIS.

Second, this study contributes to health information seeking research by using SCT to complement established health and risk information frameworks. These frameworks have advanced the understanding of health information seeking by emphasizing risk and uncertainty related mechanisms. In older adult contexts, for example, RISP has been used to explain older adults’ health information search in public health emergency contexts by focusing on health risk perception, affective responses, and information sufficiency (82). Building on this line of work, the present study adopts SCT to integrate aging-related cognitive perceptions, motivational beliefs, environmental conditions, and behavior within a unified framework. The findings suggest that older adults’ OHIS may be associated with the interplay of aging-related cognitive perceptions, self-efficacy, outcome expectations, and supportive digital environments. This extends health information seeking behavior research by highlighting the value of considering personal, motivational, and environmental factors simultaneously when explaining older adults’ digital health engagement.

Third, this study contributes to the literature on the digital divide and digital inclusion by identifying community IT culture as a contextual boundary condition. Existing research has emphasized interventions that address older adults’ individual capability deficits, such as technology training, digital literacy improvement, and family support (23, 55, 79). The present study extends this perspective by showing that the association between perceived declines in information processing speed and self-efficacy differed according to the level of community IT culture. Specifically, stronger community IT culture attenuated the negative association between perceived declines in information processing speed and self-efficacy. This finding highlights the theoretical importance of community-level digital environments in understanding older adults’ OHIS intention. Rather than viewing digital inclusion solely as a matter of improving individual capabilities, this study suggests that supportive community environments should also be incorporated into theoretical models of older adults’ digital health engagement.

### 4.3 Practical implications

First, stakeholders should focus on building older adults’ confidence (i.e., self-efficacy) in using digital technologies rather than solely on improving their technical skills. Digital training remains necessary, but given cognitive decline in older adults, family members and volunteers should understand their learning pace during instruction. They should teach step-by-step and hands-on using approaches older adults can accept. Furthermore, family members and volunteers must maintain patience, as older adults may repeatedly struggle with the same issue. When older adults demonstrate successful progress in learning, encourage them to actively explore new functions. During training sessions, digital mentors must not complete tasks for seniors. Instead, they should guide older adults step by step through operations until they can perform tasks independently. Beyond formal training, establishing peer role models can be effective. Some older adults hold negative attitudes toward digital technology, believing they are too old to adapt. Sharing successful experiences and social comparisons through role models can challenge these stereotypes, encouraging them to actively engage with new digital opportunities. Moreover, technology developers must also adapt digital tools for older adults by reducing friction in their interactions with technology. Examples include: reducing information density on web pages, providing repeatable and returnable functions, and offering clear indicators of trustworthy sources.

Second, stakeholders also need to emphasize the potential benefits of online health information-seeking behavior to enhance older adults’ external motivation. The government should encourage doctors to publish health information online, utilizing platforms such as social media and science popularization websites to share content related to disease prevention, treatment, and management. Especially when older patients seek medical care, doctors can proactively recommend reliable online healthcare resources relevant to their conditions. Platforms should also strengthen health information review mechanisms, implement professional certification for content creators, develop effective algorithms to identify health misinformation, and delete or flag questionable content to provide older adults with trustworthy and useful health resources. Additionally, peer role models prove equally effective in raising outcome expectations. When older adults observe peers similar to themselves benefiting from online health resources, they are more likely to imitate these individuals and believe they can achieve comparable outcomes.

Finally, communities can establish digital support networks for older adults, including family members, peers, and volunteers to ensure they always have access to assistance when encountering usage issues. Communities also should provide sufficient public resources for older adults, such as public internet access devices, regularly scheduled instructional workshops, and dedicated spaces for scheduled consultations. Importantly, communities must foster a positive digital culture that encourages everyone, including older adults, to participate in the digital society (e.g., accessing online health resources). Organizing digital technology learning groups for older adults and inviting them to share their experiences can eliminate the negative mindset stemming from unfamiliarity and fear of failure, particularly how they step-by-step overcome challenges and complete tasks.

### 4.4 Limitations and future research

Although this study yielded valuable findings, certain limitations warrant further exploration in future research. First, the use of self-reported measures to assess older adults’ information processing speed captures their psychological responses when searching for online health information. Future studies could employ both subjective and objective measurement methods to differentiate their respective impacts on older adults’ beliefs and behaviors. Second, as this is a cross-sectional study, it is challenging to accurately identify causal relationships between variables. Longitudinal designs can be used to accurately examine the interactions among cognitive abilities, expectation beliefs, and behaviors. Third, this study used online convenience sampling, which may limit the generalizability of the findings. Future studies should adopt more representative sampling strategies and include older adults from diverse community, rural–urban, educational, and digital access backgrounds to further validate the proposed model. Finally, this study focused solely on the moderating role of community IT culture as an environmental variable. In daily life, older adults receive support from multiple sources, such as family, peer, and volunteers. Analyzing other types of social environmental factors can provide additional evidence for developing effective intervention strategies.

## 5 Conclusion

This study examined the associations between perceived declines in information processing speed and older adults’ intention to seek online health information, situating digital health engagement barriers within concrete experiences of aging. The findings suggest that perceived declines in information processing speed are indirectly associated with lower online health information seeking intention through lower self-efficacy and less favorable outcome expectations. Community IT culture attenuated the negative association between perceived declines in information processing speed and self-efficacy, indicating that supportive community-level digital environments may help older adults maintain confidence in digital health engagement despite perceived cognitive challenges. From a digital public health perspective, these findings highlight the need to combine individual-level support, such as strengthening self-efficacy and outcome expectations, with community-level efforts to build inclusive and age-friendly digital environments.

## Data Availability

The raw data supporting the conclusions of this article will be made available by the authors, without undue reservation.
