# Supplementary material for: Perceived cognitive slowing and online health information seeking among older adults: motivational beliefs as pathways and community IT culture as a buffer
Source: Front Public Health. 2026 Jun 26;14:1875038. doi: 10.3389/fpubh.2026.1875038 (PMC13349898; doi:10.3389/fpubh.2026.1875038)
Supplement: Supplementary file 1 [file Supplementary_File_1.docx]

# Appendix A. Measurement instrument

**Perceived decline in information-processing speed**

As I get older, I experience ……

1. needing more time to analyze tasks

2. that it is more difficult for me to remember several things at once

3. that it takes me more time to organize new information

4. that learning new information take me more time

5. that I have to work harder to understand tasks that include a lot of new information

**Self-efficacy**

1. I feel confidence seeking health information on my own using smartphones

2. If I wanted to, I could easily operate smartphone to seek health information on my own

3. I can use a smartphone to seek health information even if no one is around to help me

**Outcome expectation**

1. Using a smartphone to seek health information can benefit me in managing my health

2. Using a smartphone to seek health information can improve my performance in managing my health

3. Using a smartphone to seek health information will be useful for my health

4. Using a smartphone to seek health information can enhance my effectiveness in managing my health

**Community IT culture**

1. My community always tells me the importance of smartphone in life.

2. My community uses smartphones to handle life task.

3. My community encourages me to use the smartphones in life.

**Online Health Information Seeking Intention**

1. I intend to keep seeking health information using smartphone in the future

2. I intend to seek health information on my smartphone frequently in the future

3. I am willing to seek health information using smartphone

4. I will seek related health information on my smartphone when I need

# Appendix B. Item Cross- loading

|  | PD | SE | OE | CC | OHISI |
| --- | --- | --- | --- | --- | --- |
| PD1 | ***0.759*** | -0.241 | -0.027 | -0.065 | 0.066 |
| PD2 | ***0.894*** | -0.349 | -0.182 | -0.260 | -0.064 |
| PD3 | ***0.892*** | -0.346 | -0.198 | -0.244 | -0.072 |
| PD4 | ***0.922*** | -0.403 | -0.238 | -0.260 | -0.127 |
| PD5 | ***0.918*** | -0.347 | -0.167 | -0.235 | -0.079 |
| SE1 | -0.346 | ***0.790*** | 0.304 | 0.154 | 0.199 |
| SE2 | -0.356 | ***0.898*** | 0.443 | 0.256 | 0.308 |
| SE3 | -0.316 | ***0.875*** | 0.543 | 0.263 | 0.333 |
| OE1 | -0.197 | 0.459 | ***0.876*** | 0.387 | 0.567 |
| OE2 | -0.189 | 0.423 | ***0.886*** | 0.375 | 0.562 |
| OE3 | -0.163 | 0.468 | ***0.868*** | 0.377 | 0.577 |
| OE4 | -0.155 | 0.457 | ***0.866*** | 0.347 | 0.546 |
| CC1 | -0.328 | 0.289 | 0.359 | ***0.912*** | 0.349 |
| CC2 | -0.065 | 0.158 | 0.300 | ***0.727*** | 0.434 |
| CC3 | -0.191 | 0.206 | 0.422 | ***0.882*** | 0.441 |
| OHISI1 | -0.073 | 0.277 | 0.587 | 0.457 | ***0.870*** |
| OHISI2 | -0.121 | 0.274 | 0.526 | 0.416 | ***0.824*** |
| OHISI3 | -0.026 | 0.281 | 0.513 | 0.341 | ***0.820*** |
| OHISI4 | -0.042 | 0.291 | 0.520 | 0.329 | ***0.821*** |
